# Young Adults’ Perceptions of Cigarette Warning Labels in the United States and Canada

**Published:** 2007-03-15

**Authors:** Michelle O’Hegarty, Linda L Pederson, David Nelson, Pascale Wortley, Gayane Yenokyan

**Affiliations:** Centers for Disease Control and Prevention, Office of Smoking and Health; Health Communications Branch, National Center for Chronic Disease Prevention and Health Promotion, Office on Smoking and Health. Linda L. Pederson was with Northrop Grumman Information Technology Business Computer Associates, Atlanta, Ga, when the study was done; Health Communications Branch, National Center for Chronic Disease Prevention and Health Promotion, Office on Smoking and Health; National Center for Immunization and Respiratory Disease, Centers for Disease Control and Prevention, Atlanta, Ga; Department of Epidemiology, Johns Hopkins University, Baltimore, Md

## Abstract

**Introduction:**

For the past 20 years, there have been no changes to the text-only cigarette warning labels in the United States. During this same time period, other countries placed large graphic warning labels on cigarette packages. The purpose of this study was to investigate the reactions of U.S. young adult smokers and nonsmokers aged 18 to 24 years to Canadian cigarette label text and graphic warnings. The study focused on determining their perceptions and the potential impact of Canadian labels on smoking, and study participants were asked for suggestions for modifications of U.S. cigarette warning labels so they would be effective for smoking deterrence and cessation.

**Methods:**

During January and February 2002, 11 focus groups consisting of 54 smokers and 41 nonsmokers were conducted in the Detroit metropolitan area. Current smokers were defined as those who had smoked a cigarette within the past 30 days. Participants were asked about their knowledge and perceptions of current U.S. cigarette warning labels and their impressions of Canadian cigarette warning labels.

**Analysis:**

A content analysis and a word index were applied to the transcripts of all focus groups to identify and clarify themes and domains that appeared in group discussions and to compare results across different groups.

**Results:**

Focus group participants reported that Canadian cigarette warning labels were more visible and informative than U.S. cigarette warning labels. Messages perceived to be relevant to smokers were considered effective. Education level did not appear related to how participants responded to warning labels. There were some differences for warning labels that had sex-specific messages.

**Discussion:**

Warning labels are one component of comprehensive tobacco control and smoking cessation efforts. Stronger warnings on cigarette packages need to be part of a larger U.S. public health educational effort.

## Introduction

Health warning labels on cigarette packages were introduced in the United States in 1965 to inform consumers about the health risks associated with tobacco use. The 1965 Federal Cigarette Labeling and Advertising Act required that the statement, *Caution: Cigarette smoking may be hazardous to your health*, be placed in small print on one side panel of every cigarette pack (1). In 1981, the Federal Trade Commission (FTC) concluded that the health warning did not provide sufficient information to consumers about the health hazards of smoking and that the message was overexposed, outdated, abstract, and not personally relevant to consumers (2).

In 1984, Congress enacted the Comprehensive Smoking Education Act, which required rotation of the following four black and white text messages on the side of cigarette packages:

1. *Surgeon General's warning: Smoking causes lung cancer, heart disease, emphysema, and may complicate pregnancy.*
2. *Quitting smoking now greatly reduces serious risks to your health.*
3. *Smoking by pregnant women may result in fetal injury, premature birth, and low birth weight.*
4. *Cigarette smoke contains carbon monoxide* (3).

For the past 20 years, there have been no changes to U.S. cigarette package warning labels.

Several other countries, including Canada, Australia, Thailand, South Africa, Singapore, and Poland, have mandated stronger health warnings on cigarette packages (4-11) by requiring the addition of graphic images and detailed statistical information regarding the health risks of tobacco use and information about how to quit smoking. Graphic warnings are now present on cigarette packages in five countries: Canada, Brazil, Singapore, Thailand, and Australia. In 1989, Canada had text-only warning labels that covered 20% of cigarette packages (12). In 2000, Canada passed new regulations enlarging cigarette warning labels to 50% of the front and back of the cigarette package. These labels included text, graphic color photos, and information on toxic substances (13-15).

In the United States, Canada, and Australia, research was previously conducted to examine major elements of warning labels and to increase how much they are noticed and their perceived believability (4,5,16-25). Recommendations for improving cigarette labels included increasing the size of cigarette warnings (16,19-23); adding color images (16,17,19-21); using strong, personalized messages (17,20,23); using plain packaging that does not include logos, colors, or text that may distract the consumer from warnings (20,24); and including tobacco ingredients on packages (25). Evaluations of new warning labels in Australia and Canada show they attract the attention of smokers (26,27), increase awareness of the health hazards of smoking (5,27,28), increase beliefs about risks associated with smoking (4,27,29),  and decrease cigarette consumption (4,5,27). An important outcome of this research was the information that some smokers who had attempted to quit smoking previously said the new warnings played a part in motivating them to try again to quit (9).

The Office on Smoking and Health at the Centers for Disease Control and Prevention (CDC) contracted with Opinion Research Corporation (ORC) Macro to conduct focus groups in Oakland County, Michigan, part of the Detroit metropolitan area, to examine knowledge, attitudes, and predicted behavior toward Canadian warning labels among young U.S. adults. We sought to determine what these young adults perceived as the potential impact of explicit graphic warning labels as compared with the current U.S. black and white warning text labels, and we wanted to obtain suggestions for modifying Canadian cigarette labels for use in the United States. We also wanted to know whether listing ingredients on cigarette packages would prevent initiation of smoking or provide reasons for smokers to quit. We included only young adults aged 18 to 24 years because of the potential impact of warning labels on this age group and the idea that young adults who do not smoke or only smoke occasionally may be influenced by label information. We were also interested in learning how responses to labels differed on the basis of respondents' sex and educational status.

## Methods

### Focus group recruitment and selection

We submitted this study to the CDC institutional review board (IRB), and it met CDC criteria for IRB exemption. Participants were recruited by phone calls made by ORC Macro from their telephone center database of 35,000 households in Oakland County, Michigan. Since the database contained relevant demographic information, it was possible to recruit individuals with specific characteristics for this study, and we selected African Americans and whites in numbers proportional to their representation in the 2000 census for Oakland County.

Of 572 people contacted, 211 (37%) refused to participate, and 201 (35%) were ineligible. Of the 160 potential eligible participants, 54 smokers and 41 nonsmokers participated in our focus group sessions. Another 65 potential participants did not arrive for their scheduled session or were not needed for focus group discussions. Those who agreed to participate received $50 for their time and travel expenses.

During January and February 2002, there were 11 focus group sessions with eight to nine participants in each group. Four groups had participants who were college students or recent graduates, and seven groups had participants who were not in college and were not recent graduates. Participants were self-identified as white or African American, aged 18 to 24 years, and residents of the Detroit metropolitan area. The facility where focus group sessions were held was equipped with an observer viewing room with one-way mirrors. A moderator trained in conducting focus groups guided each group discussion.

Participants were segmented by smoking status, sex, and education level and were divided into two groups based on educational level, *in college or recent graduate *and *not in college or recent graduate* (Table) to create homogeneous groups (30,31). The *not in college or recent graduate* group included those participants with a high school degree or GED (general educational development) certificate and no plans to attend college in the next year. Smokers and nonsmokers were separated to assess their similarities and whether their perceptions of cigarette warning labels were different. Guides* for the moderator differed slightly based on the smoking status of participants.

Current smokers were defined as those who had smoked a cigarette within the previous 30 days. Nonsmokers were defined as those who did not currently smoke, had not smoked in the previous 6 months, and who did not plan on smoking in the next 6 months.

### Focus group sessions

Participants completed a prediscussion information sheet* that included questions about age, education, employment, and martial status. Questions about personal smoking behaviors and smoking among close friends and family members were included. Information sheets revealed that participants in the *not in college or recent graduate* group tended to smoke more frequently than those in the *in college or recent graduate* group. Five to seven participants in the *not in college or recent graduate* group reported smoking every day in the previous 30 days, but only two participants in the *in college or recent graduate* group reported this level of smoking frequency. Of the 54 current smokers, 20 reported smoking 11 to 20 cigarettes per day, and 15 reported smoking 2 to 5 cigarettes per day.

Before the 1.5-hour focus group session discussion began, the moderator reviewed the informed consent form signed by each participant. During the sessions, both smokers and nonsmokers were asked if they had ever seen warnings on cigarette packages and were shown six Canadian cigarette warning labels that were available in 2002 (Figure).

**Figure.**

**Figure:**
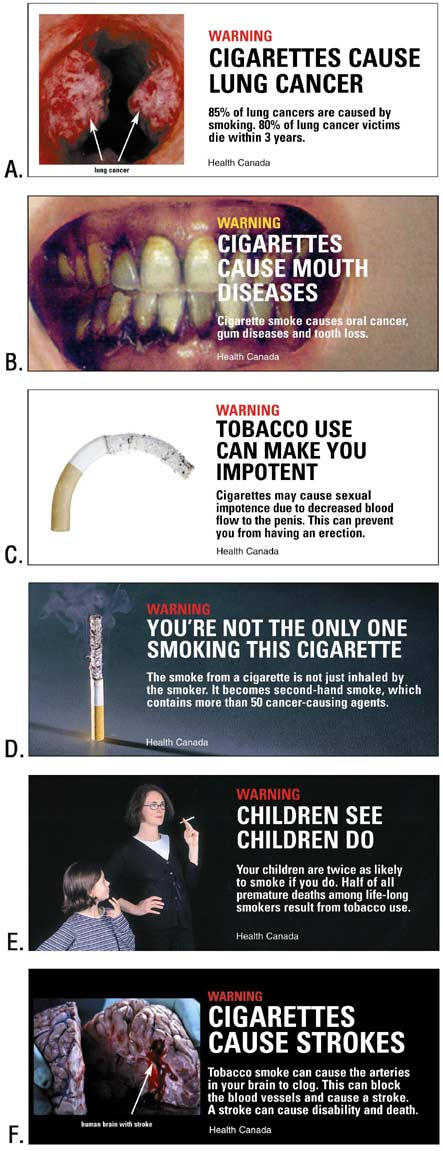
Six Canadian health warning labels placed on cigarette packages sold in Canada, 2002.

After participants viewed the Canadian cigarette label health warnings, discussion questions focused on participants' reactions and perceptions of the visual and contextual format of these labels. The moderator also asked participants about the value of listing cigarette ingredients on cigarette packages. Only smokers were asked whether warning labels would motivate them to quit and whether they thought warnings would deter young people from smoking.

## Analysis

We conducted a content analysis evaluation of all focus group transcripts to assist in creating a replicable analytical tool and to validate data (32). Content analysis was used to clarify group discussion themes and to compare results from different focus groups. We used a word index to identify transcript themes. Each word was listed, a tally was made of the number of times each word was used, and the location of each word in the transcript was noted. Themes were identified, labeled, and categorized directly by participants or were identified by the study team. There was little disagreement about themes, and the few disagreements that did occur were discussed and easily resolved.

## Results

### Focus group participants' impressions of U.S. cigarette warning labels

A majority of focus group participants recalled seeing U.S. warning labels on cigarette packages and could recall some of the messages. Smokers indicated that they did not pay attention to the U.S. labels.

*You see them, but I don't think you read them. I don't, at least.*

Male smoker, *In College or Recent Graduate*

*If you've got the cigarette in hand, you're not thinking about it. You're really not. You're just thinking, 'I need to take care of this urge.'*

Female smoker, *Not in College or Recent Graduate*

Both men and women focus group participants questioned the credibility of U.S. warning labels on cigarette packages because of the language used. In the words of one smoker, "It says 'Warning: *may* cause heart disease or birth complications.' It's not definite." Even before seeing the Canadian labels, several participants mentioned that the Canadian government cares more about its citizens because its warning labels give factual information and do not use phrases like *may cause cancer* instead of *will cause cancer*. There were no obvious differences by sex or level of education in reactions to U.S. labels.

### Focus group participants' impressions of Canadian cigarette warning labels

Before participants were shown pictures of the Canadian cigarette warning labels, the moderator asked if they had seen the warning labels previously and what their general impressions were of the labels. Even though the focus groups were held in Detroit and were close to Canada, only a few participants said they had previously seen current Canadian cigarette packages with their graphic warning labels.

*It puts a visual picture in your head to go along with words that you've been hearing. So here's something like you can see, not just the words behind it. It's something you can see, so it's going to affect you maybe a little bit more.*

Male smoker, *In College or Recent Graduate*

*American cigarettes — I've read the warning labels, and they all say the same thing. But Canada — that makes me think a little bit more because they're so blunt about it. They really say — smoking is going to kill you. That's on the label.*

Female smoker, *Not in College or Recent Graduate*

After all focus groups saw the six Canadian cigarette warning labels, participants indicated the Canadian labels were more likely to be seen and were more informative than U.S. cigarette warnings. As one man, a smoker who was in the *Not in College or Recent Graduate* group, said, "I think if our [U.S.] warning labels were more like those [Canadian], people would read them once in a while. It would be better if [American warnings] had a message that [gave] percentages and facts."

Warnings perceived to be most helpful by both smokers and nonsmokers were those that combined strong visual images and compelling facts, such as label A (Cigarettes Cause Lung Cancer) and label F (Cigarettes Cause Strokes). Many participants noted that they did not know about the smoking-stroke relationship. Potential life-threatening risks from smoking were of greatest concern to smokers. Warning label B regarding oral diseases was more meaningful among nonsmokers than smokers. Many participants thought the image was the mouth of a heavy smoker, not a mouth with oral cancer and gum disease, and questioned the credibility of the warning. Many participants who were smokers considered this warning an example of a scare tactic that did not realistically present health risks associated with tobacco use and stated it would not encourage them to quit smoking. One woman participant who was a smoker and college educated stated, "Perhaps this is really far?fetched because it's not like a common problem, you know. I work at a dentist's office, and I've never seen somebody with that problem.*"*

Nonsmokers considered the gum disease warning to be most influential among young people who do not smoke or among those who have just begun smoking. Among both smokers and nonsmokers, the secondhand smoking messages in warning labels D and E had the weakest impact of all six labels. Smokers thought the message on label D (*You're Not the Only One Smoking This Cigarette*) was targeting people who did not smoke or smokers who live with people who have health problems. Another reaction encountered was that the visual image of the burning cigarette appealed to some smokers.

Warning label E, depicting a child with a mother who is smoking and the message *Children See Children Do,* did not attract participants' attention, probably because few of them had children. Some female nonsmokers thought the message was truthful.

Because the majority of participants were unaware that smoking could cause impotence (warning label C), they were skeptical about whether this message would encourage smokers to quit. Male smokers acknowledged that this type of message would make them rethink smoking but indicated the warning would not cause them to quit unless they were experiencing a problem with impotence.

*I mean, this would probably make a guy think twice, but it probably wouldn't stop him from smoking. He'd probably forget about it in a couple of days. The next time he gets an erection, he'd probably forget about it.*

Male smoker, *In College or Recent Graduate*

*I think it's a good bar topic. I can see my friends sitting around laughing at this. I mean, it might be true, but it's not going to make like my fiancé quit smoking."*

Female smoker, *Not in College or Recent Graduate*

The message on warning label C did not include factual information to back up the claim that c*igarettes may cause sexual impotence due to decreased blood flow to the penis.* Information such as the age group that could be affected or the percentage of men who experienced this side effect would strengthen the effect of this warning. Without this additional information, participants were doubtful about the veracity of the message. This warning was perceived by all participants as targeting a limited audience. Interestingly, women thought the message targeted men, and men thought women or older men would be most affected by the message.

There were no noticeable perception and opinion differences between participants in the *in college or recent graduate* group and participants in the *not in college or recent graduate* group in regard to warning labels on cigarette packages. 

### Participants' recommended changes to U.S. cigarette warning labels

Participants' major recommendation about the U.S. labels was to add graphic images to cigarette packaging so U.S. warning labels would be similar to those in Canada. Some participants suggested that smokers might be influenced by a label comparing a healthy lung, a lung from someone who had smoked for 5 to 10 years, and a lung from someone with lung cancer. Additional recommendations included 1) increasing the size of warning messages; 2) adding factual, realistic information to warning labels; and 3) using bright colors and bold text for message information. Several participants suggested that warning labels with messages about secondhand smoke should state the potential dangers of such smoke to children and other family members who do not smoke. Including images of children on warning labels with messages on secondhand smoke may be a successful mechanism to discourage smokers from smoking around others or to get them to quit.

*I think to make this more effective, you'd have like a group of kids. I'll never smoke around kids. Like I don't even want them around because I know there's secondhand smoke. But if you do put a picture of kids, I think it would be a lot more effective, because you'd start thinking about that. Like you don't want to hurt them if they're not choosing to smoke.*

Male smoker, *In College or Recent Graduate*

When smokers were asked about whether cigarette ingredients should be added to U.S. cigarette packages, many smokers said yes. Some participants mentioned learning about tar, nicotine, and carbon monoxide because of messages from the American Legacy Foundation's truth campaign, but they did not know about specific health risks for these ingredients. When asked whether listing ingredients would discourage people from smoking, one participant mentioned that it might help smokers cut down on the number of cigarettes smoked if they could see and compare levels of tar in various cigarette brands.

## Discussion

Our findings are consistent with those from several qualitative and quantitative studies conducted between 1990 and the early 2000s in the United States, Canada, and Australia. These studies examined major elements of cigarette warning labels to determine what would increase the amount of attention they received and their believability (4,5,16-24). Findings from previous studies and from our study show that increasing warning label size (16,19-23) and adding color images (16,17,19-21) can affect the attention a message receives and its believability. There were no differences in perceptions of the effectiveness of warning labels between participants in the *in college or recent graduate* group and the *not in college or recent graduate* group. Differences by sex were noted, primarily for warning labels that had sex-specific messages (e.g., some women participants could relate to the woman in the warning label *Children See Children Do* because they were parents themselves). If parents do not want their children to smoke, warning labels could serve as reminders that parental behavior may influence the smoking behavior of their children (33).

A 2001 study that evaluated Canadian cigarette package warnings among adults aged 18 years and above found that among both smokers and nonsmokers the warnings most effective at discouraging smoking were those portraying a diseased mouth and those showing a lung tumor (9). Previous studies and our study show that messages relevant to smokers (e.g., those showing what lung cancer looks like) are likely to be effective. In our study, messages not relevant or believable to viewers (e.g., impotence, gum disease) were less likely to motivate smokers to quit smoking.

A Canadian study by Koval et al using survey questions found that Canadian young adult smokers aged 20 to 24 years who had been exposed to Canadian tobacco warning labels were less likely to believe that some warning labels would make people their age less likely to smoke (34). Our study reported similar findings. Some men in the study were skeptical about the effectiveness of labels in encouraging smokers to quit, such as warnings about mouth disease, smoking and impotence, and children seeing parents smoke as well as about the effectiveness of warning labels in encouraging smokers to quit smoking.

Study participants' recommendations about ways to increase awareness of U.S. cigarette warning labels include 1) using plain packaging that does not show tobacco industry logos, colors, or text to distract consumers from the warning (20,24) and 2) including tobacco ingredients on packages (35). Recommendations from participants are consistent with the 2003 World Health Organization Framework Convention on Tobacco Control guidelines for cigarette warning messages, which recommend that they cover 50% or more of a cigarette package display area and include graphics (5,23).

Canada and Australia have had success at increasing awareness of smoking health hazards (5, 27,28) and motivating smokers to quit smoking (9) by modifying the health warnings on cigarette packages. Evaluations of cigarette health warning labels in Australia and Canada show the labels attract the attention of smokers (26,27), increase awareness and knowledge of smoking health hazards and risks (4,5,27-29), and decrease cigarette consumption (4,5,27). Some smokers who had previously attempted to quit smoking reported the Canadian warning labels would motivate them to try again (9).

Some limitations need to be considered in interpreting the results of this study. All focus groups were held in Oakland County, Michigan, and different results might be obtained elsewhere. Focus groups were limited to young adults aged 18 to 24 years who were selected from ORC Macro's database of 35,000 households in Oakland County. Had other focus groups been assembled (e.g., middle-aged adults, smokers contemplating quitting), the outcomes might have been different. In addition, of the 160 eligible participants, 65 did not arrive at the facility for their scheduled session or were not needed for a group discussion. It is not known whether findings would have been the same if these individuals had participated in discussions.

In this study we examined tobacco-related knowledge and attitudes among young adults in the United States and compared their perceptions of how U.S. and Canadian cigarette warning labels would affect smoking behavior. We found some differences by sex in their reactions to labels, and these differences support efforts to design warning labels that target different groups. Stronger warnings on U.S. cigarette packages that include graphic images and factual messages may help consumers make more informed decisions about using tobacco products. Warning labels need to be part of a larger public health education effort and incorporated into antismoking campaigns so they can reinforce antismoking messages and provide information about the health risks of tobacco use (33). Stronger warnings on U.S. cigarette packages can enhance public health efforts for tobacco prevention and control.

## Figures and Tables

**Table:** Focus Groups by Smoking Status, Sex, and Education Level, Detroit, 2002

Groups: Sex and Education Level	Smokers, No. Groups	Nonsmokers, No. Groups
**Men**		
In college or recent graduate	1	1
Not in college or recent graduate^a^	2	2
**Women**		
In college or recent graduate	1	1
Not in college or recent graduate^a^	2	1

a Participants with a high school degree or GED (general educational development) certificate and no plans to attend college in the next year.
